# Distinguishing PTSD, Complex PTSD, and Borderline Personality Disorder: A latent class analysis

**DOI:** 10.3402/ejpt.v5.25097

**Published:** 2014-09-15

**Authors:** Marylène Cloitre, Donn W. Garvert, Brandon Weiss, Eve B. Carlson, Richard A. Bryant

**Affiliations:** 1 National Center for PTSD, Veterans Affairs Palo Alto Health Care System, Palo Alto, CA, USA; 2 Department of Psychiatry and Child & Adolescent Psychiatry, New York University Medical Center, New York, USA; 3 Department of Psychiatry and Behavioral Sciences, Stanford University School of Medicine, Palo Alto, CA, USA; 4 School of Psychology, University of New South Wales, Sydney, NSW, Australia

**Keywords:** Complex PTSD, posttraumatic stress disorder, Borderline Personality Disorder, WHO, ICD-11

## Abstract

**Background:**

There has been debate regarding whether Complex Posttraumatic Stress Disorder (Complex PTSD) is distinct from Borderline Personality Disorder (BPD) when the latter is comorbid with PTSD.

**Objective:**

To determine whether the patterns of symptoms endorsed by women seeking treatment for childhood abuse form classes that are consistent with diagnostic criteria for PTSD, Complex PTSD, and BPD.

**Method:**

A latent class analysis (LCA) was conducted on an archival dataset of 280 women with histories of childhood abuse assessed for enrollment in a clinical trial for PTSD.

**Results:**

The LCA revealed four distinct classes of individuals: a Low Symptom class characterized by low endorsements on all symptoms; a PTSD class characterized by elevated symptoms of PTSD but low endorsement of symptoms that define the Complex PTSD and BPD diagnoses; a Complex PTSD class characterized by elevated symptoms of PTSD and self-organization symptoms that defined the Complex PTSD diagnosis but low on the symptoms of BPD; and a BPD class characterized by symptoms of BPD. Four BPD symptoms were found to greatly increase the odds of being in the BPD compared to the Complex PTSD class: frantic efforts to avoid abandonment, unstable sense of self, unstable and intense interpersonal relationships, and impulsiveness.

**Conclusions:**

Findings supported the construct validity of Complex PTSD as distinguishable from BPD. Key symptoms that distinguished between the disorders were identified, which may aid in differential diagnosis and treatment planning.

There has long been debate about whether Complex Posttraumatic Stress Disorder (Complex PTSD) is distinct from Borderline Personality Disorder (BPD) comorbid with PTSD. Part of the difficulty in this evaluation has been the lack of clear and consistent characterization of Complex PTSD. The World Health Organization (WHO) Working Group on the Classification of Stress-Related Disorders has proposed the inclusion of Complex PTSD as a new diagnosis related to but separate from PTSD (Maercker et al., 2013). Both of these disorders are viewed as distinct and separate from BPD. An emerging and accumulating empirical literature is demonstrating consistent and clear differences between ICD-11 PTSD and Complex PTSD. In addition, it is important to determine the construct validity of Complex PTSD as empirically distinct from BPD particularly among those with a trauma history. This investigation evaluated whether ICD-11 Complex PTSD could be distinguished from DSM-IV BPD in a treatment-seeking population of women with childhood abuse.

The WHO proposed that the development of ICD-11 be guided by the principle of clinical utility. Characteristics of clinical utility include the organization of disorders that are consistent with clinicians’ mental health taxonomies, that contain a limited number of symptoms so that they can be easily recalled and used in the field, and that are based on distinctions important for management and treatment (Reed, 2010). The distinction between ICD-11 PTSD and Complex PTSD are consistent with these guidelines (see Cloitre, Garvert, Brewin, Bryant, & Maercker, 2013; Maercker et al., 2013). For example, ICD-11 PTSD is construed as a fear-based disorder and symptoms are limited to and consistent with fear reactions and consequent avoidance and hypervigilence. In contrast, Complex PTSD has been described as typically associated with chronic and repeated traumas and includes not only the symptoms of PTSD but also disturbances in self-organization reflected in emotion regulation, self-concept and relational difficulties (see Cloitre et al., 2013) a symptom profile that has been demonstrated as associated with prolonged trauma (Briere & Rickards, 2007).

Three studies have found evidence supporting the validity of the ICD-11 PTSD versus Complex PTSD distinction (see Table 1 for description of the diagnoses). Recently, in order to evaluate whether PTSD and Complex PTSD could be empirically distinguished from each other, Cloitre and colleagues (2013) performed a latent profile analysis (LPA) on assessment data from 302 treatment-seeking individuals with diverse trauma histories, ranging from single events (e.g., 9/11 attacks) to sustained exposures (e.g., childhood or adult physical and/or sexual abuse). The results were consistent with the ICD-11 formulation for Complex PTSD, with the best fitting LPA model delineating three classes of individuals: (1) a Complex PTSD class, with high levels of both PTSD symptoms as well as disturbances in self-organization related to affect regulation problems, negative self-concept, and relational difficulties; (2) a PTSD class, with high levels of PTSD symptoms but relatively low on the disturbances in self-organization that define Complex PTSD; and (3) a class relatively low on symptoms of both PTSD and Complex PTSD. Notably, these identified classes were identical when including an additional 86 participants with BPD, providing further support for the stability of the identified classes. Cloitre et al. (2013) also found that chronic trauma was more predictive of Complex PTSD than PTSD and that Complex PTSD resulted in significantly greater functional impairment than PTSD.

**Table 1:** Symptom profile for each diagnosis and items used in the LCA analyses

Symptoms for each diagnoses			
	
ICD-11 PTSD	ICD-11 CPTSD	DSM-IV BPD	Items
Re-experiencing	Re-experiencing		
Flashbacks	Flashbacks		CAPS 1: Unwanted memories of the event
Nightmares	Nightmares		CAPS 2: Recurrent distressing dreams of the event
Avoidance	Avoidance		
Thoughts	Thoughts		CAPS 6: Avoid thoughts, feelings or conversations
People, places, activities	People, places, activities		CAPS 7: Avoid activities, places, or people
Sense of threat	Sense of threat		
Hypervigilance	Hypervigilance		CAPS 16: Being especially alert constantly on guard
Startle	Startle		CAPS 17: Strong startle reactions
	Emotion regulation		
	Anger		BSI 13: Temper outburst that you could not control
	Hurt Feelings		BSI 20: Your feelings being easily hurt
	Negative self-concept		
	Worthless		BSI 50: Feeling of worthlessness
	Guilty		BSI 52: Feelings of guilt
	Interpersonal problems		
	Not close		BSI 44: Never feeling close to another person
	Feel disconnected		CAPS 10: Feeling distant or cut off from other people
		Frantic	SCID-II 90: Frantic efforts to avoid abandonment
		Unstable relationships	SCID-II 91: Unstable and intense relationships with alternating extremes of idealization and devaluation
		Unstable sense of self	SCID-II 92: Markedly and persistently unstable sense of self
		Impulsiveness	SCID-II 96: Impulsiveness that is potentially self-damaging
		Self-harm	SCID-II 97: Recurrent suicidal behavior, gestures, or threats, or self-mutilating behavior
		Mood changes	SCID-II 99: Affective instability due to reactivity to mood
		Empty	SCID-II 100: Chronic feelings of emptiness
		Temper	SCID-II 101: Frequent displays of anger, constant anger, recurrent physical fights
		Paranoid/dissociation	SCID-II 104: Transient, stress-related paranoid ideation or severe dissociative symptoms

Elklit, Hyland, and Shevlin (2014) replicated the findings of Cloitre and colleagues (2013), performing a latent class analysis (LCA) on three separate samples of trauma-exposed Danish individuals who experienced primary traumas of bereavement, sexual assault, and physical assault. The investigators found that the LCA model with the best fit for each sample consisted of three classes of individuals identical to those identified by Cloitre et al. (2013). Lastly, Knefel and Lueger-Schuster (2013) performed confirmatory factor analysis (CFA) on data from 226 Austrian adults who had experienced institutional abuse, defined as physical, sexual or emotional abuse by individuals representing institutions responsible for the care of children (i.e., Catholic Church, foster care). Results indicated that individuals diagnosed with Complex PTSD had experienced significantly longer exposure to traumatic situations and that the theoretically derived CFA model demonstrated good model fit. Overall, these studies provide substantial support for the construct validity of Complex PTSD across international samples of individuals exposed to diverse traumatic events, demonstrating that it is a diagnostic entity distinct from PTSD and supporting the recommendations of the ICD-11 proposal.

The argument that Complex PTSD is an amalgam of PTSD and BPD has been built on reports of the relatively high comorbidity between PTSD and BPD. For example, using data from the National Epidemiologic Survey on Alcohol and Related Conditions (NESARC), a nationally representative sample of United States population, Pagura and colleagues (2010) found that 24% of individuals with lifetime PTSD also met criteria for BPD, 30% of individuals with BPD also met criteria for lifetime PTSD, and 2% had comorbid PTSD and BPD. Also utilizing NESARC data, Grant and colleagues (2008) found that 29% of individuals who currently met criteria for PTSD in the past 12 months also met criteria for lifetime BPD, and 32% of individuals with lifetime BPD met criteria for 12-month PTSD. In clinical samples, the rates of comorbidity are higher. PTSD patients are reported to have BPD comorbidity ranging from 37 to 68% (Heffernan & Cloitre, 2000; Zlotnick, Franklin, & Zimmerman, 2002) and conversely, among BPD patients 25–58% are diagnosed with comorbid PTSD (Golier et al., 2003; Harned, Rizvi, & Linehan, 2010; Zanarini et al., 1998).

Despite these high rates of comorbidity, the key clinical features of Complex PTSD and BPD differ and lead to different treatment implications, a consequence of significance when considering the clinical utility of diagnostic formulation. Complex PTSD includes PTSD symptoms and, accordingly, treatment highlights the amelioration of the trauma memory as a key goal (Cloitre et al., 2011). In contrast, the key impairing features of BPD are self-injurious and suicidal behaviors, and treatment activities focus on the resolution of these behaviors (e.g., Linehan, 1993). There are several other ways in which the profile of Complex PTSD differs from that of BPD. First, it should be noted that BPD does not require a traumatic stressor for diagnosis and PTSD symptoms may or may not be present. Rather, BPD is characterized by fear of abandonment, shifting self-image or self-concept, shifting idealization and devaluation in relationships, and frequent impulsive and suicidal behaviors (see Table 1). In Complex PTSD, as proposed in ICD-11, the fear of abandonment is not a requirement of the disorder, self-identify is consistently negative rather than shifting and relational disturbances highlight chronic avoidance of relationships rather than sustained chaotic engagement. While emotion regulation difficulties are central to both Complex PTSD and BPD, their expression is quite different. In Complex PTSD they predominantly include emotional sensitivity, reactive anger and poor coping responses (e.g., use of alcohol and substances). In BPD, some of the preceding may be observed, but the criteria, perhaps the defining characteristics of the disorder, include suicide attempts and gestures as well as self-injurious behaviors, events which occur much less frequently in complex forms of PTSD than in BPD samples (e.g., Zlotnick et al., 2002). Given these identified differences in diagnostic formulation and their treatment implications, empirical evaluation of Complex PTSD and BPD in a symptom-by-symptom manner is important.

The purpose of the current study was to determine whether the symptoms endorsed by women seeking treatment for childhood abuse form classes that are consistent with diagnostic criteria for PTSD, Complex PTSD, and BPD (see Table 1). We hypothesized that analyses would reveal at least three distinct classes of individuals with the following symptom profiles: (1) high levels of ICD-11 PTSD symptoms *but not* symptoms of Complex PTSD or BPD, (2) high levels of Complex PTSD symptoms (PTSD plus emotion regulation, negative self-concept and interpersonal problems) *but not* BPD symptoms; (3) high levels of BPD symptoms with an admixture of trauma-related symptoms (e.g., PTSD, CPTSD symptoms).

## Methods

### Participants and procedures

The data for these analyses were obtained from an archival set of measures administered as part of an assessment procedure for a randomized controlled trial for PTSD related to childhood abuse (*n*=310) (see Cloitre et al., 2010). The data are a subset of individuals for whom complete measures of PTSD, BPD, general psychopathology, and functional impairment were available (*n*=280).

Participants had a mean age of 37.13 (SD = 10.86) years. The entire sample was female and 40% identified as Caucasian (40.4%, *n*=113), followed by African-American (26.4%, *n*=74), Hispanic (18.6%, *n*=52), Asian (3.9%, *n*=11), other (8.6%, *n*=24), and unreported (2.1%, *n*=6). Marital status of the sample was as follows: 54.3% (*n*=152) reported being single, married (11.1%, *n*=31), divorced or separated (16.1%, *n*=45), living with a significant other (15.7%, *n*=44), widowed (0.7%, *n*=2), and unreported (2.1%, *n*=6). College graduation or completion of some college was reported by 64.3% (180), postgraduate education was reported by 24.3% (68), high school graduation or some high school was reported by 9.3% (26), and education level was unavailable for 2.1% (6). The majority of participants reported some employment with 41.4% (116) employed full-time (35+ hours per week) and 23.9% (*n*=67) employed part-time (<35 hours per week).

Frequency of traumas were as follows: childhood sexual abuse (CSA) (65.1%), childhood physical abuse (CPA) (80.8%), neglect (46.4%), emotional abuse (80.4%), 35.9% were not living with their mother before the age of 18, adulthood sexual assault (ASA) (49.6%), and adulthood physical assault (APA) (26.0%). All individuals had experienced either CPA or CSA.

### Measures

#### Clinician Administered PTSD Scale

The Clinician Administered PTSD Scale (CAPS) for DSM-IV PTSD is a well-validated clinician administered interview (see Weathers, Keane, & Davidson, 2001) that evaluates the presence and severity of the 17 DSM-IV PTSD symptoms over the past month with separate five-point scales for frequency (ranging from 0 = “never” to 4 = “almost daily”) and intensity (ranging from 0 = “none” to 4 = “extreme”). The CAPS items used for current analyses are listed on Table 1. An item with a frequency score of 1 (“once or twice in the past month”) or higher and an intensity score of 2 (“moderate”) or higher was considered positive for that symptom following guidelines suggested by Weathers et al. (2001).

#### Brief Symptom Inventory

The Brief Symptom Inventory (BSI) is a 53-item self-report psychological symptom inventory with nine primary symptom dimensions. The measure assesses how much a problem bothered or distressed a person using a 5-point Likert scale ranging from 0 = “not at all” and 4 = “extremely”. The BSI has shown high convergent and construct validity (Derogatis & Melisaratos, 1983). The BSI items used for the current analyses are listed on Table 1. An item score of 2 (“moderately”) or higher was considered positive for that symptom.

#### Structured Clinical Interview-II Borderline Personality Disorder module

The Structured Clinical Interview for Axis II Disorders (SCID-II) DSM–IV BPD Module has nine items where a score of 1=absent or false, 2=subthreshold and 3=threshold or true (First, Spitzer, Gibbon, & Williams, 1994). The items used for the current analyses are listed on Table 1. An item score of 3 was considered positive for that symptom.

#### Social Adjustment Scale-Self Report

The Social Adjustment Scale-Self Report (SAS-SR; Weissman & Bothwell, 1976) was utilized to measure functional impairment. The SAS-SR consists of 42 Likert-type items, which assess the level of functioning over the past 2 weeks for six domains: work, social and leisure activities, relationships with extended family, role as a marital partner, parental role, and role within the family unit. A mean score can be calculated for each of the six domains, as well as one overall mean score, based on the total number of items relevant to the responder. Higher scores indicate greater impairment. The SAS-SR has demonstrated strong psychometric properties among community and clinical samples (e.g., Weissman & Bothell, 1976).

### Statistical analyses

#### Latent class analysis

The model fit for the optimal number of classes that were examined were the Lo-Mendell-Rubin adjusted likelihood ratio test (LMR-A), the bootstrap likelihood ratio test (BLRT), the Bayesian Information Criterion (BIC), the Sample-Size Adjusted BIC (SSA-BIC), and the Akaike Information Criterion (AIC). In a simulation study, the BLRT was shown to outperform the LMR-A and the BIC (among other measures of model fit) in selecting the number of classes (Nylund, Asparouhov, & Muthen, 2007). Since there is not a clear-cut decision rule on how to select the best fitting model, we ranked ordered the importance of fit indices as follows: BLRT, BIC, SSA-BIC, AIC, and then the LMR-A. The general practice of LCA is to test the fit of a two-class model and systematically increase the number of classes until adding more classes is no longer warranted. The LMR-A and the BLRT compare the fit of the specified class solution to models with one less class. A *p*<0.05 suggests that the specified model provides a better fit to the data relative to the model with one less class. A total of 21 symptoms (coded dichotomously as present or not) were used in the LCA, 6 representing the ICD-11 PTSD symptoms, 6 representing the self-organization symptoms unique to Complex PTSD), and 9 representing the SCID-II1 symptoms of BPD (see Table 1). The LCA models were estimated using robust maximum likelihood method with 400 initial stage random starts and 80 final stage optimizations to determine if the best log-likelihood value was obtained and replicated. Finally, 50 bootstrap draws were used in the BLRT.

#### Descriptive statistics

Chi-square tests and ANOVAs were performed to assess differences in sociodemographic characteristics, trauma history, and symptom severity across the classes identified in the LCA. Descriptive statistics were computed based on valid (non-missing) data.

## Results

### 

#### Latent class analysis

The fit indices of the different class models examined are shown in Table 2. The two-class model yielded a significant LMR-A and BLRT result at *p*<0.05. The three- and four-class models both yielded a significant BLRT result at *p*<0.05, but not a significant LMR-A result. A five-class model was examined, but the best log-likelihood value was not replicated, and it was not considered for the final model, as the *p*-value may not be trustworthy due to local maxima. The four-class model did not have the lowest BIC value, but it was selected over the two- and three-class models because it was the model with the largest number of classes that had a significant and trustworthy BLRT result, had the lowest SSA-BIC value, and had the lowest AIC value of the models considered. The three- and four-class models were examined closely, as they both could have legitimate arguments for being selected. However, based on all of the fit indices examined and on the interpretability of the symptom profiles of the classes (consistent with study hypotheses), the four-class model was selected.

**Table 2:** Latent class models and fit indices

Model	Log-likelihood	BIC	SSA-BIC	AIC	Entropy	LMR-A *p*-value	BLRT *p*-value
2 classes	−3523.010	7288.315	7151.965	7132.020	0.781	0.029	<0.001
3 classes	−3433.024	7232.310	7026.199	6996.048	0.817	0.066	<0.001
4 classes	−3382.025	7254.278	6978.406	6938.051	0.808	0.401	<0.001
5 classes	−3338.211	7290.613	6944.981	6894.421	0.848	0.639	<0.001^a^

*Note*. BIC, Bayesian Information Criterion; SSA-BIC, Sample-Size Adjusted BIC; AIC, Akaike Information Criterion; LMRA-A, Lo-Mendell-Rubin adjusted likelihood ratio test; BLRT, bootstrap likelihood ratio test.

a The best log-likelihood value was not replicated in 32 out of 50 bootstrap draws. The *p*-value may not be trustworthy due to local maxima.

**Fig. 1:**
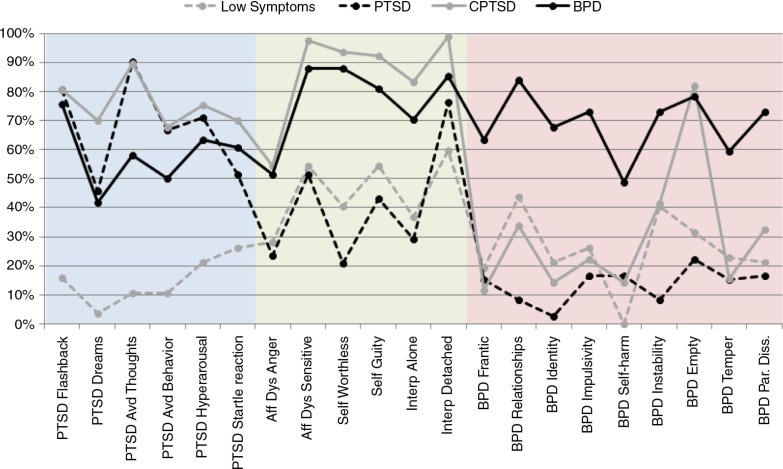
Symptom endorsement of Complex PTSD and BPD items by class.

The pattern of symptom endorsement for all four classes is presented in Fig. 1. The four classes were compared on the 21 symptoms that were used to determine class membership in order to provide descriptive labels for each class. The Low Symptom class had relatively low levels of all symptoms across all domains. The PTSD class had generally high levels of PTSD symptoms, but relatively low levels of self-organization and BPD symptoms. The CPTSD class had high levels of PTSD and self-organization symptoms, but relatively low levels of BPD symptoms. The BPD class had a high percentage of BPD symptoms as well as self-organization disturbances and PTSD symptoms.

The average probability of latent class membership in the four-class model was acceptable at 0.91 for the Low Symptom class, 0.92 for the PTSD class, 0.87 for the CPTSD class, and 0.91 for the BPD class, which implies acceptable discrimination between the classes. An acceptable entropy value probability of 0.81 lends support to this result by suggesting adequate latent class separation. Overall, 20.4% (*n*=57) of participants were classified into the Low Symptom class, 25.7% (*n*=72) into the PTSD class, 27.5% (*n*=77) into the CPTSD class, and 26.4% (*n*=74) into the BPD class.

#### Sociodemographic and trauma history characteristics

ANOVA and Chi-square analyses were performed to assess differences in sociodemographic characteristics, trauma history, and symptom severity across the classes identified in the LCA. Results shown in Table 3 indicate that the four classes did not differ by age, ethnicity, or employment status. The classes also did not differ in the rates of types of childhood or adulthood interpersonal traumas, with the exception that CSA was reported more frequently in the CPTSD class than in the Low Symptom and BPD classes. Total number of different types of traumatic experiences did not differ across classes.

**Table 3:** Demographic and trauma characteristics of the classes

Characteristics	Class 1 Low Symptom *n*=57	Class 2 PTSD *n*=72	Class 3 CPTSD *n*=77	Class 4 BPD *n*=74	Significance test
Age	37.91 (10.17)	36.21 (10.64)	36.95 (10.42)	37.63 (12.10)	NS
Race (% white)	44.4%	42.9%	33.8%	45.2%	NS
Employed (FT or PT)	66.0%	71.0%	68.4%	64.4%	NS
CSA	54.5%	66.7%	80.5%	55.4%	*p*=0.003 3>1, 4
CPA	80.0%	82.9%	81.8%	78.4%	NS
Neglect	34.5%	47.1%	54.5%	45.9%	NS
Emotional abuse	78.2%	75.7%	79.2%	87.8%	NS
Any childhood abuse	98.2%	98.6%	97.4%	95.9%	NS
ASA	38.9%	46.4%	57.1%	52.7%	NS
APA	29.6%	18.8%	32.5%	23.3%	NS
Any adult assaults	53.7%	55.9%	72.7%	65.8%	NS
Both child and adult events	51.9%	55.9%	71.4%	63.5%	NS

*Note*. All tests were Chi-square tests with 3 degrees of freedom.

#### Symptom characteristics

The rates of probable disorders (ICD-11 PTSD, ICD-11 CPTSD and BPD) as well as the percent of endorsed symptom characteristics for all 21 symptoms across the four classes are presented in Table 4. Overall, 53.9% (*n=*151) had a PTSD diagnosis, 38.2% (*n=*107) had a CPTSD diagnosis, and 29.3% (*n=*82) had a BPD diagnosis. Of those with a BPD diagnosis, majority also had a PTSD diagnosis (54.9%, *n=*45) and 45.1% (*n=*37) had a CPTSD diagnosis. In the Low Symptom class, no one met criteria for either PTSD or CPTSD while 12% met criteria for BPD. The most common symptoms were unstable relationships, mood changes and feeling empty. Of individuals in the PTSD class, 68% met criteria for PTSD, but only 19.4% met criteria for CPTSD and 1.4% met criteria for BPD. Of individuals in the CPTSD class, 77.9% met criteria for CPTSD but only 7.8% met criteria for BPD. Of those in the BPD class, 91.9% met DSM-IV BPD diagnosis. Overall, the DSM-IV BPD diagnosis fit very few of the individuals in the CPTSD (7.8%) class but the large majority of the BPD class (91.9%).

**Table 4:** Frequencies of endorsement for ICD-11 PTSD, CPTSD, and DSM-IV BPD items

Symptoms	Class 1 Low symptoms (*n*=57)	Class 2 PTSD (*n*=72)	Class 3 CPTSD (*n*=77)	Class 4 BPD (*n*=74)	Significant pairwise post-hoc comparisons
ICD-11 PTSD diagnosis	0.0%	68.1%	80.5%	54.1%	2, 3, 4>1 3>4
Re-experiencing					
Flashbacks	15.8%	80.6%	80.5%	75.7%	2, 3, 4>1
Nightmares	3.5%	45.8%	70.1%	41.9%	2, 3, 4>1 3>2, 4
Avoidance					
Thoughts	10.5%	90.3%	89.6%	58.1%	2, 3, 4>1 2, 3>4
People, places, or activities	10.5%	66.7%	67.5%	50.0%	2, 3, 4>1
Sense of threat					
Hypervigilance	21.1%	70.8%	75.3%	63.5%	2, 3, 4>1
Startle	26.3%	51.4%	70.1%	60.8%	2, 3, 4>1
ICD-11 CPTSD diagnosis	0.0%	19.4%	77.9%	44.6%	3>1, 2,4 4>1, 2
Affect regulation problems					
Angry	28.1%	23.6%	54.6%	51.4%	3, 4>1, 2
Hurt feelings	54.4%	51.4%	97.4%	87.8%	3, 4>1, 2
Negative self-concept					
Worthless	40.4%	20.8%	93.5%	87.8%	3, 4>1, 2
Guilty	54.4%	43.1%	92.2%	81.1%	3, 4>1, 2
Interpersonal problems					
Not close	36.8%	29.2%	83.1%	70.3%	3, 4>1, 2
Feel disconnected	59.7%	76.4%	98.7%	85.1%	3, 4>1 3>2, 4
DSM-IV BPD diagnosis	12.3%	1.4%	7.8%	91.9%	4>1, 2, 3
Frantic	19.3%	15.3%	11.7%	63.5%	4>1, 2, 3
Unstable relationships	43.9%	8.3%	33.8%	83.8%	4>1, 2, 3 1, 3>2
Unstable sense of self	21.1%	2.8%	14.3%	67.6%	4>1, 2, 3 1>2
Impulsiveness	26.3%	16.7%	22.1%	73.0%	4>1, 2, 3
Self-harm	0.0%	16.7%	14.3%	48.7%	4>1, 2, 3 2, 3>1
Mood changes	40.4%	8.3%	41.6%	73.0%	4>1, 2, 3 1, 3>2
Empty	31.6%	22.2%	81.8%	78.4%	3, 4>1, 2
Temper	22.8%	15.3%	15.6%	59.5%	4>1, 2, 3
Paranoia/dissociation	21.1%	16.7%	32.5%	73.0%	4>1, 2, 3

*Note*. All tests were Chi-square tests with 3 degrees of freedom, and the significance of all tests was *p*<0.01; reported significant pairwise post-hoc comparisons used an adjusted *p*-value using the Bonferroni method.

A review of the individual items indicates that, consistent with the graphic depiction provided in Fig. 1, the BPD class had a lower rate of endorsement of the ICD-11 PTSD symptoms across all items as compared to the CPTSD class. The rates were significantly lower for nightmares and avoidance of trauma-related thoughts. Endorsement of the individual items reflecting disturbances in self-organization by the BPD class members was similar to that of the CPTSD class. However, only 44.6% of the BPD class met criteria for CPTSD suggesting that individuals did not consistently endorse the CPTSD symptoms across all three categories of disturbance (emotion dysregulation, negative self-concept, interpersonal problems) sufficient to complete the CPTSD profile. Indeed, individuals in the BPD class were more likely to meet criteria for PTSD (54%) than CPTSD.

The CPTSD class had significantly lower endorsement of all the BPD symptoms than the BPD with the exception of feelings of emptiness. The CPTSD class was more similar to the PTSD class in regard to endorsement of BPD symptoms. The two classes did not differ from each other on the BPD symptoms in seven out of nine symptoms, with the exception of unstable relationships and mood changes, which were both endorsed at higher rates in the CPTSD class than the PTSD class. Notably, almost half of the BPD class members endorsed self-harm/suicidal behaviors while this behavior was not endorsed by anyone in the Low Symptoms class and by a relatively low and equal proportion in the PTSD and CPTSD classes (16.7 and 14.3%, respectively).

#### Functional impairment

Functional impairment was greatest in the BPD (M=2.34, SD=0.43) and CPTSD class (M=2.31, SD=0.39) relative to the PTSD (M=2.76, SD=0.48) and Low Symptom (M=2.71, SD= 0.52) classes. The BPD and CPTSD classes did not differ significantly from each other in functional impairment (*p*=0.920). Similarly, the PTSD and Low Symptom classes did not differ significantly from each other in functional impairment (*p*=0.983).

#### BPD symptoms as indicators of risk for BPD versus CPTSD diagnosis

The salience of each of the BPD symptoms as a “marker” of being in the BPD class compared to the CPTSD class was evaluated. Relative risk (RR) was computed for each symptom (see Table 5). RR provides the likelihood that a person positive on a particular symptom will be in the BPD class relative to the CPTSD class. Each of the BPD symptoms was much more likely to be associated with the BPD class versus the CPTSD class, except for emptiness. The strongest symptom predictors of class were: frantic about abandonment, unstable relationships, unstable sense of self and impulsiveness.

**Table 5:** Relative risk of SCID-II BPD items—comparing BPD versus CPTSD classes

BPD symptoms	Relative risk	95% CI
Frantic	2.95*	2.10, 4.15
Unstable relationships	3.70*	2.18, 6.26
Unstable sense of self	3.07*	2.14, 4.42
Impulsiveness	3.04*	2.04, 4.55
Self-harm	2.10*	1.56, 2.83
Mood changes	2.04*	1.37, 3.04
Empty	0.90	0.61, 1.32
Temper outbursts	2.49*	1.80, 3.45
Dissociation	2.46*	1.65, 3.68

CI=Confidence Interval.

* *p*<0.01.

Interpretation example: Individuals positive on the Frantic symptom had a 2.95 times greater risk of being in the BPD class than those without the Frantic symptom.

## Discussion

Overall, the findings showed that the patterns of symptoms endorsed formed classes that are consistent with diagnostic criteria for PTSD, Complex PTSD, and BPD. The LCA identified four distinct classes of individuals within a treatment-seeking sample: a Low Symptom class that was relatively low in all measured symptoms; a PTSD class that was high in symptoms of PTSD but relatively low in self-organization symptoms and symptoms of BPD; a Complex PTSD class that was high in symptoms of PTSD and self-organization symptoms but relatively low in symptoms of BPD; and a BPD that was high in symptoms of BPD with additional symptoms of PTSD and CPTSD. These distinct classes demonstrated acceptable discrimination. Additionally, these classes did not differ in demographic variables (e.g., age, ethnicity, employment status) or total number of traumas experienced. These findings provide empirical support that the symptom profiles endorsed by individuals with Complex PTSD and BPD result in distinguishable subgroups of trauma-exposed individuals.

While the individuals in the BPD reported many of the symptoms of PTSD and CPTSD, the BPD class was clearly distinct in its endorsement of symptoms unique to BPD. The RR ratios presented in Table 5 revealed that the following symptoms were highly indicative of placement in the BPD rather than the CPTSD class: (1) frantic efforts to avoid real or imagined abandonment, (2) unstable and intense interpersonal relationships characterized by alternating between extremes of idealization and devaluation, (3) markedly and persistently unstable self-image or sense of self, and (4) impulsiveness. Given the gravity of suicidal and self-injurious behaviors, it is important to note that there were also marked differences in the presence of suicidal and self-injurious behaviors with approximately 50% of individuals in the BPD class reporting this symptom but much fewer and an equivalent number doing so in the CPSD and PTSD classes (14.3 and 16.7%, respectively). The only BPD symptom that individuals in the BPD class did not differ from the CPTSD class was chronic feelings of emptiness, suggesting that in this sample, this symptom is not specific to either BPD or CPTSD and does not discriminate between them.

It should be noted that the endorsement of the CPTSD symptoms related to self-organization disturbances was high among members of the BPD class. It may be that the presence emotion regulation problems does not distinguish CPTSD and BPD, although the severity and type might, i.e., suicidality, self-injurious behavior are characteristic of BPD not CPTSD. Alternatively, it may be that the BSI is not optimal as a measure of self-organization disturbances to provide differential diagnosis of CPTSD versus BPD. The BSI tracks symptoms only for the past 2 weeks, and thus chronicity of symptoms was not assessed. Members of the BPD class may have some but not all of the CPTSD symptoms and may vary in their endorsement of symptoms across time while the symptoms as endorsed by the CPTSD class would be expected to be chronic and stable. This interpretation is consistent with the data from the SCID-II questions where items highlighting lack of stability were strongly endorsed by the BPD but not the CPTSD and PTSD class members.

Overall, the findings indicate that there are several ways in which Complex PTSD and BPD differ, consistent with the proposed diagnostic formulation of CPTSD. BPD is characterized by fears of abandonment, unstable sense of self, unstable relationships with others, and impulsive and self-harming behaviors. In contrast, in CPTSD as in PTSD, there was little endorsement of items related to instability in self-representation or relationships. Self-concept is likely to be consistently negative and relational difficulties concern mostly avoidance of relationships and sense of alienation. Lastly, a comment on the Low Symptom class is deserved. The class seems comprised of individuals who have very low endorsement of PTSD symptoms but somewhat higher endorsements on disturbances in self-organization. These symptoms may reflect the presence of subsyndromal BPD or symptoms resulting from a mix other Axis I disorders (Bipolar Disorder, Major Depression). Future studies, which evaluate Axis I disorders and provide subsyndromal diagnoses, will help decipher the nature of this class.

The distinct symptom profiles characterizing CPTSD and BPD lead to different treatment considerations. The focus of treatment for BPD concerns reduction of life-interfering behaviors such as suicidality and self-injurious behaviors, a reduction in dependency on others and an increase in an internalized and stable sense of self (e.g., Dialectical Behavior Therapy, Linehan, 1993). In contrast, treatment programs for CPTSD focus on reduction of social and interpersonal avoidance, development of a more positive self-concept and relatively rapid engagement in the review and meaning of traumatic memories (e.g., Cloitre et al., 2006). Duration of treatment for each disorder and attention to the termination phase are different as well. Experts in the treatment of BPD have noted that the termination of treatment is a time of risk for relapse and symptom exacerbation (see Harned & Linehan, 2008). The end of therapy may provoke feelings of abandonment, destabilize identity and lead to impulsive and self-injurious behaviors. The DSM guidelines for BPD recommend treatment duration of at least 1 year (American Psychiatric Association, 2013). A treatment course of a year or more may allow for demonstrated success in reduction of life-interfering behaviors, the reinforcement and routinization of effective emotion management skills and a carefully planned end to treatment. While the recommended duration of treatment for Complex PTSD has not yet been established, it seems likely be shorter than for BPD given the presence of a stable sense of self and relative absence of substantial risk for self-injurious behaviors and suicidality, but longer than that for PTSD, given the greater number and diversity of symptoms (see Cloitre et al., 2012).

Growing attention to patient-centered care, which emphasizes the patient's specific symptoms, needs and preferences will hopefully facilitate the development of treatment programming that neither under-treats nor over-treats the patient. The proposed spectrum of diagnoses moving from PTSD to CPTSD and BPD may provide a foundation for developing algorithms of type of interventions and duration of care that meets the needs of patients with symptom profiles that differ in clinically significant ways.

A number of limitations of the current study are worth noting. First, the sample consisted of a treatment-seeking sample with a history of childhood interpersonal trauma. Replication of results is necessary with samples that are more representative of populations in clinical and community settings. Future studies should include samples with greater diversity in types of trauma as well as diversity in the exposure to traumatic stressors. Secondly, the data used in the analyses are from a secondary source and do not represent the ideal basis for evaluating ICD-11 PTSD and Complex PTSD symptoms. The Structured Interview for Disorders of Extreme Stress (SIDES, Pelcovitz et al., 1997), a structured clinician driven measure which assesses many of the symptoms of Complex PTSD was not available in this data set. Also, the time duration for which the symptoms were assessed differed across measures and thus did not allow consistency in the assessment of the chronicity or variability of the symptoms endorsed. However, the study results, which provide evidence of qualitative differences between the CPTSD and BPD symptom profiles, suggest the importance of developing empirically validated measures of ICD-11 PTSD and CPTSD and their comparison to BPD in a variety of clinical and epidemiological samples.

## Conclusion

This study identified four distinct classes of individuals who have experienced trauma, supporting the proposed distinction between Complex PTSD and BPD. Key symptoms that differentiate BPD from Complex PTSD were identified. These findings conform to ICD-11's proposed distinction between the diagnoses. They also point to the merits of pursuing the construct of CPTSD as a separate clinical entity from PTSD and BPD. However, to achieve this agenda it is important that empirically validated measures of CPTSD be developed for standardized assessment of the construct in relation to PTSD and BPD. Given that that there are efficacious treatments for CPTSD (Cloitre et al., 2010) and BPD (e.g., Linehan, 1993), and these approaches vary in important ways, it is useful for clinicians to be able to differentiate between these presentations.
